# Explaining Consumer Intentions for Foods with Antioxidant Properties: Predictors of Choice and Purchase Barriers

**DOI:** 10.1155/2021/9971425

**Published:** 2021-07-12

**Authors:** Rinat Fatkullin, Natalya Naumenko, Natalia Popova, Alena Ruskina, Irina Kalinina, Irina Potoroko

**Affiliations:** School of Medical Biology, The Department of Food and Biotechology South Ural State University (NIU), Russia

## Abstract

Today, food products not only should serve the source of main nourishment but also must minimize the risk of negative impact on the human body. Products enriched with antioxidants can be referred to such category. For food producers, the development of products “for health” is usually connected with significant investment, whereas the final success of innovative products does not always meet the expectations. The greatest part of such products is withdrawn from the market during the first year. It is important for manufacturers to learn consumer behavior in order to ensure sale growth and a stable market position. The purpose of this study was to study consumer reception of products containing antioxidants. (It is important to conduct market research to identify the needs of buyers in order to maintain a stable position in the market. In order to study this issue, we analyzed the consumer perception of foods with antioxidant properties.) We studied the consumer perception of products with antioxidant properties with regard to choice predictors and barriers for purchasing, which finally determine the success of the product on the market. For this purpose, we conducted a survey of 721 consumers of the South Urals. The results of the statistical analysis done with the help of SPSS proved that South Ural consumers in general are ready to purchase products containing antioxidants. Besides, producers must bring information concerning the real value of the product and win the consumer trust and confidence as far as these are the main predictors determining the choice for purchasing the products containing antioxidants. Misunderstanding of the role antioxidants play in the human body may lead to perception of risk concerning consumption of such products and rejection of the purchase.

## 1. Introduction

Currently, there is a significant increase in the interest of consumers living in territories with a high technogenic load in preventive food products that have the maximum effect on health. It is known that in combination with adverse environmental factors, poor nutrition contributes to the emergence and development of many diseases. The problem of the annual growth of noncommunicable diseases is most typical for the regions of high environmental risk, which include the territory of the Southern Urals, as a center of industrial production and ferrous metallurgy.

The complex ecology of the environment determines a serious problem of receipt and retention in the human body of products of industrial enterprises, including heavy metals and radionuclides. The analysis of NCDs that are prevalent in the Southern Urals: anemia, asthma, type II diabetes, oncology, etc., shows the influence of the environmental situation on the prevalence of diseases and the frequency of their manifestations [1–3]. This indicates the increasing role of preventive measures for noncommunicable diseases. One of these tools can be food products enriched with biologically active food ingredients, including antioxidant action. The role of foods with antioxidants in the diet of the population has especially increased due to the situation associated with COVID-19, when a high level of stress determines the activation of oxidative processes and associated negative consequences for the human body.

At the same time, the supply of health products, including antioxidant products, at the trade enterprises of the Southern Urals is very limited. Among the products-sources of antioxidants, bakery and fermented milk products with fruit and berry additives, products from sprouted grains, soft drinks on a fruit, and berry basis predominate. However, in the overall structure of the products sold, health products occupy no more than 15% and often do not stand out in a separate product line. According to [4], one of the reasons for this situation is the reluctance of retailers to take on additional financial risks that may be associated with a poor consumer response to new products.

[5–7] expressed that the production of enriched, functional, and specialized food products is inevitably associated with expensive technological and research investments. According to [8–11], commercial distribution of products is complicated by the absence of both the internationally recognized concept of these products and the still inactive regulatory system in particular countries. Based on the literature [12–14], differences between the concepts and definitions of functional foods, including dietary supplements and nutraceuticals, in the United States, Japan, Russia, and the European Union increase the difficulties of the food industry in marketing functional foods. On the one hand, the creation of a product requires large investment from the idea stage to its introduction into the consumer market. On the other hand, according to [15], consumers' perception of such innovative products can be very unpredictable. This happens due to the fact that the ultimate success of innovative products largely depends on which factors will influence consumer perceptions and what requirements and expectations consumers will have for these products.

Based on the literature [16–18], currently, world statistics is such that 70-90% of new products aimed at improving human health leave the market the first two years after their launch. Newer studies [19–22] have shown that the discrepancy between the expectations and/or needs of consumers and the real benefits of functional foods can contribute to a high risk of product failure in the market. According to [23] in order to increase the chances of success in functional foods market, consumer acceptance has been identified as the decisive factor. Thus, in recent years a relevant amount of papers have reported empirical studies based on primary data collection in order to analyze consumer attitudes towards functional foods.

Many studies have focused on consumer awareness and acceptance of functional foods [24–26], attitudinal profiles and motivations to use functional foods [17, 27–31], and factors affecting willingness to pay for functional foods [24–26]. A common result of these studies is that consumer acceptance of functional foods is far from being unconditional. Accordingly, in the past three decades, many researchers have introduced models for predicting human behavior, such as the Theory of Planned Behavior. The model of the Theory of Planned Behavior proven to [32–34] successfully predict behavioral intention of individuals was applied in the current study as a framework in order to predict consumers' purchase intention of food products with antioxidant properties. In order to study this topic, we carried out consumer perception analysis of food products with antioxidant properties, using survey format. We set the task of establishing predictors of consumer choice in favor of products with antioxidant properties, as well as the barrier factors of their purchase.

### 1.1. Research Framework and Hypotheses

As a tool for solving the task, we used factor and regression analysis using the program product SPSS.

The main objectives of the study were

- to identify consumer attitudes towards “health products” in general
- to identify consumer attitudes towards products with antioxidant properties
- to establish the level of consumer understanding of the role and functions of antioxidants for human health
- to establish the level of consumer confidence in the manufacturers of “health products”
- to establish a list of food products most preferred for antioxidant enrichment

**Figure 1:**
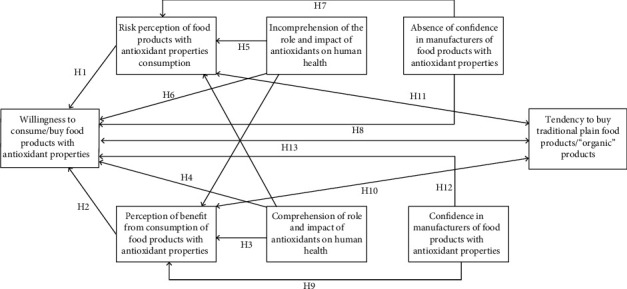
Working model and expected correlations (Hi: hypotheses).

At the first stage, we made up a working model of the hypothesis and determined the expected correlations to establish the factors influencing consumers' choice of enriched products, including products with antioxidant properties (Figure 1).

#### 1.1.1. H1 and H2: The Perceptions of the Risk and Benefits of the Consumption of Products with Antioxidant Properties Were Put Forward as the Main Hypotheses

Despite the fact that quantitative research on consumer opinion regarding functional, enriched, special-purpose products is rather scarce, the analysis of qualitative research regarding consumer risks suggests some assumptions. According to [35–39], consumers participating in surveys, as a rule, are divided into two groups: those who prefer traditional products which are free of any additives and those who see the benefits of having enriching additives in food products. Taking it into consideration, it is reasonable to assume that the risk and benefit are important factors for consumers to make a decision about purchasing healthy food products, including those with the antioxidants added.

Expectations that nutritional additives are beneficial increase the consumption of a product, while perceptions of risk reduce it. Thus, in our working model, the first two hypotheses are that consumer preferences for foods with antioxidants are negatively associated with risk perceptions (H1) and positively associated with perceptions of the antioxidants benefits (H2).

#### 1.1.2. H3–H6: Comprehension or Incomprehension of the Antioxidants' Role and Functions for Human Health

Scientific knowledge of action, functions, and the role of antioxidants in human health is of great importance for consumers of food products enriched with antioxidants. Published studies indicate that “ignorant” consumers perceive the risk of taking food with some additives much higher than knowledgeable consumers do. Thus, the hypotheses H3 and H4 are that comprehension of the functions and the role of antioxidants for human health is negatively associated with the perception of risk (H3) and positively associated with the acceptance of the product as a whole (H4). Incomprehension of functions and the role of antioxidants for human health are negatively associated with the perception of benefits (H5) and the acceptance of the product as a whole (H6).

#### 1.1.3. H7–H10: Confidence or Lack of Confidence in Manufacturers of Products with Antioxidant Properties

Another important factor known in the scientific literature and research of consumer behavior risks is that consumers make decisions about buying a “health product,” based on their confidence in its manufacturers. Confidence plays a key role in new products and sophisticated food technology. Thus, hypotheses H7 and H8 are that confidence in product manufacturers is positively related to the benefit perception (H7) and the acceptance of the food product as a whole (H8). At the same time, lack of confidence in food manufacturers is positively related to risk perception (H9) and is negatively related to the food product acceptance as a whole (H10).

#### 1.1.4. H11–H13: Preference for Products of Traditional Composition and Natural Free Of Additives Products

Finally, in recent years, the role of the product naturalness is essential in shaping consumer preferences. Organic products are considered to be safer and healthier, while products containing artificial and synthetic additives are associated with a sense of risk for consumers. For some consumers, “no additives” or “organic” is an important characteristic of a food product. There are studies indicating that a number of consumers are extremely conservative in shaping their own consumer behavior and prefer products of traditional composition to the enriched ones even if the latter lack potential benefits. Consequently, the hypotheses H11–H13 lie in the fact that the preference for products of traditional composition or natural products with “no additives” increases the perception of risk (H11) and reduces the perception of benefit (H12) and the acceptance of a product with antioxidant additives as a whole (H13).

## 2. Methods

### 2.1. Data Collection and Profile of Respondents

To study consumer perception of products containing antioxidant additives and establish the main factors forming it, we worked out a questionnaire and conducted a consumer survey.  Table 1 provides a brief description of the study.

Survey participants were randomly chosen by a convenience sampling [40]. Demographic and education characteristics of the survey participants are presented in Table 2.

Thus, more women (61.0%) than men (39.0%) took part in the survey. The participants were mostly young people (66.0% were 45 years old or younger) and highly educated (44.0% had at least higher education).

### 2.2. Research Questionnaire

For the correlation analysis, we worked out a questionnaire made of 30 questions relating to one of the categories of factors (a sample of the questionnaire is presented below):

I. Willingness to buy/take products enriched with antioxidants (useful additives).

II. Risk perception from the use of the antioxidant products.

III. Benefits perception consuming antioxidant products.

IV/V. Comprehension/incomprehension of the antioxidants action in the human body.

VI/VII. Confidence/lack of confidence in enriched food producers.

VIII. The preference for organic products and with no additives.

5-6 questions of both direct and reverse order were formulated for each category of factors. Survey participants were asked to answer the questions using the Likert 5-point scale:

5 – I agree;

4 – I rather agree;

3 – Do not know;

2 – I rather disagree;

1 – I disagree.

Questions categories:

I. Willingness to buy/consume food products enriched with antioxidants (useful additives).

1. I buy some food products which contain antioxidants (useful additives).
2. I think it is important to pay attention to the presence of useful substances in a food product.
3. I think that antioxidants are healthful.
4. People are too concerned about their health.
5. I think that food products with antioxidants are healthful.
6. I am willing to buy antioxidant-enriched foods at an affordable price.

II. Risk perception from the use of antioxidant products

1. I think antioxidants can be harmful for health.
2. I believe that most food additives are harmful for health.
3. I think antioxidants can cause allergies.
4. Products with antioxidant additives make me worried.
5. I think that products with antioxidants (useful additives) are safe.

III. Perception of the benefits of using antioxidant products

1. I think food should be healthy.
2. I believe that the value of antioxidants is too exaggerated.
3. The benefits of antioxidant enriched food products are obvious.
4. Eating antioxidant food products is beneficial to me.
5. I think antioxidant products are essential.

IV/V. Comprehension/incomprehension of antioxidants action

1. I believe that antioxidants slow down the aging process.
2. I believe that antioxidants interfere with the oxidation processes in the body.
3. Most antioxidants are artificial.
4. Antioxidants slow down food spoilage.
5. I think antioxidant foods can be consumed without limitation.

VI/VII. Confidence/lack of confidence in enriched food producers

1. No harmful substances are used in food products.
2. Everything that the food products contain is reflected on the label.
3. Only verified additives are used in food products.
4. Manufacturers monitor strictly the amount of useful additive.
5. Manufactures of enriched food products take into account the norms of nutrients consumption.

VIII. Preference for organic products

1. I believe that the most useful products are organic products without additives.
2. I am willing to buy organic products for a higher price.
3. The more organic the product is, the more nutrients it contains.
4. When buying, I pay attention to the naturalness of the product.
5. I feel good when I use organic foods.

## 3. Results

All statistical calculations were performed in SPSS software version 20.0. The simple correlation analysis performed at the first stage showed the presence of a large number of significant correlations of variables not only with the dependent variable, but also with each other. To reduce the dimensionality and to represent these variables as factors that are independent from each other, a factor analysis was conducted [41]. The factor analysis was carried out in 3 stages. At the first stage, we obtained data on the number of factors into which the analyzed variables are grouped (exploratory factor analysis). Then, the number of factors was determined on the basis of a cumulative analysis of the obtained values of the Kaiser criterion and inflection points of the scree plot. At the third stage, we performed the confirmatory factor analysis, which confirms that the number of factors, which we chose, and the rotation describe our data quite well. At the first stage, the method of “principal components” was applied as a method for factor selecting.  Table 3 shows the results.

The cumulative analysis of the Kaiser criterion values obtained and the inflection points of the Scree plot allowed to establish 6 independent factors that were used later for the regression analysis.

The statistical analysis obtained a rotated factor matrix (method of factors discriminating: maximum likelihood method; method of rotation: varimax with Kaiser normalization). Analysis of the factor load allowed us to establish the following list of factors:

1. Perception of antioxidants as beneficial additives (benefit perception)
2. Comprehension of the antioxidants' role in a human body
3. Preference for organic products
4. The perception of antioxidants as harmful additives (risk perception).
5. Incomprehension or misconception of the antioxidants' role for human health
6. Confidence in antioxidant manufacturers

At the next stage, we performed a regression analysis and provided its results in 5 models. The values were obtained for each of the 5 models and ranged from 0.326 for the first model to 0.428 for the fifth model. The analysis of the obtained values of the *R*-squared allowed us to choose model 5 as the most suitable.

According to model 5, the statistically significant predictors of buying foods with antioxidants are factors 1, 2, 4, 5, and 6. The ANOVA analysis allowed us to establish the beta coefficients for each of the predictors, which made it possible to estimate the role of each of the factors. At the next stage, we obtained the correlation coefficients between the factors (Po Spearman), which are presented in Table 3.

**Figure 2:**
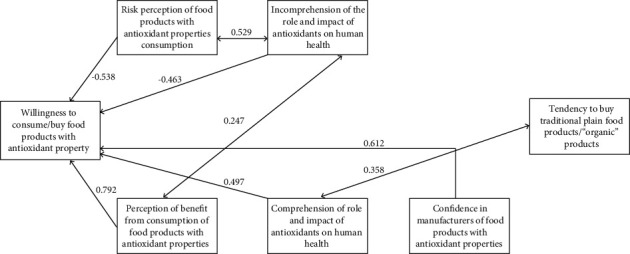
Estimation model with standardized correlation coefficients (*p* < 0.05).

The obtained correlation coefficients were correlated with the hypothesis model set-up at the initial stage of research (Figure 2).

## 4. Discussion

Thus, we can identify two most significant factors as the main predictors that determine the consumer choice of products with antioxidants. They are the expectation of benefits from the use of these products and the confidence in manufacturers; the correlation coefficients are 0.792 and 0.612, respectively. A smaller contribution to the formation of consumer choice in favor of products with antioxidants is a factor in comprehension of antioxidants' role in the human body; the correlation coefficient is 0.497.

According to the analysis, the main barrier factors include the perception of consumers' risk of using products with antioxidants and lack of comprehension or ignorance of the role and effects of antioxidants on the human body (the correlation coefficients are -0.538 and -0.463, respectively).

It is worth noting that a statistically significant correlation between the factors of comprehension of the antioxidants' role and the expectations of benefits from the products with antioxidants consumption has not been established.

However, we established a negative correlation at the level of -0.377 between the factor of incomprehension of the role and action of antioxidants in the human body and the consumers' choice of products with antioxidants. Moreover, incomprehension of antioxidants' role had a positive correlation with both the expectation of benefits and the risks of consuming foods with antioxidants (correlation coefficients are 0.247 and 0.529, respectively).

The established correlation between the lack of understanding of the role and action of antioxidants in the human body and the expectation of risk indicates a lack of consumer knowledge about antioxidants. For some respondents, the term antioxidant was associated with undesirable chemical additives rather than with useful food ingredients. The results indicate the need to popularize information about the benefits of plant. We have not established any statistically significant correlation between the factors of “preference for organic foods” and consumers' choice of food products with antioxidants.

## 5. Conclusions

Thus, the results obtained in the statistical data processing in the opinion poll with the participation of more than 700 people let us set forth a number of strategies aimed at promoting products with antioxidants to the consumer market:

1. Manufacturers have to provide more information about the benefits of natural antioxidants and products containing them for human well-being and health. Studies have shown that comprehension of the antioxidants' role and function in the human body is important for the formation of consumer preferences in favor of products with antioxidant properties. It is important to provide information about the benefits of such products; i.e., manufacturers are faced with the task of forming an image of a “better-for-you product”
2. Manufacturers of products with antioxidants need to gain the confidence of consumers. The results of the study show that confidence in the manufacturer has a significant positive impact on the attitude to the product and purchase intention. According to the Ajzen theory [42], confidence in the manufacturer when forming purchase intentions is largely identified as a safety factor, which in some cases may be more significant than comprehension of the product benefits

In general, it seems important to make such products more visible in the markets and more effectively sell their health benefits to consumers. This can further facilitate the differentiation of functional foods from other generic foods. In addition, it seems necessary to prove the scientific value of such products, given that it is still unclear how effective health-related products really are.

The limitations of this study are mainly related to the factors analyzed. However, taking all these factors into account is beyond the scope of our study. Thus, the results of our research should be considered rather exploratory and preliminary. Further research may expand our study to more of the factors being analyzed and extend to other territories.

## Figures and Tables

**Table 1:** Characteristics of the study.

Indicator	Indicator characteristics
1	2
Survey period	2019–2020
The language of the survey	Russian
Target audience	Consumers of South Urals
Data collection	Online survey, personal interview
Number of the surveyed	721 people
Software used for the survey analysis	SPSS 20.0

**Table 2:** Characteristics of respondents participating in the survey.

Characteristics		Amount	
Criterion	Subcriterion	Nominal	%
1	2	3	4
Sex	Men	281	39
	Women	440	61
Age	Under 30	281	39
	30-45	195	27
	46-65	159	22
	Over 65	86	12
Education	Secondary	173	24
	Specialized secondary	231	32
	Higher	274	38
	Scientific degree	43	6

**Table 3:** Correlation coefficients of factors (SPSS 20.0).

	*Y* ^∗^	REGR factor score 1 for analysis 2	REGR factor score 2 for analysis 2	REGR factor score 3 for analysis 2	REGR factor score 4 for analysis 2	REGR factor score 5 for analysis 2	REGR factor score 6 for analysis 2
*Y*							
REGR factor score 1 for analysis 2	0.792^∗∗∗^						
REGR factor score 2 for analysis 2	0.497^∗∗^	0.084					
REGR factor score 3 for analysis 2	0.026	-0.067	0.358^∗∗^				
REGR factor score 4 for analysis 2	-0.538^∗∗∗^	-0.063	0.111	0.073			
REGR factor score 5 for analysis 2	-0.463^∗∗^	0.247^∗∗^	0.182	0.114	0.529^∗∗∗^		
REGR factor score 6 for analysis 2	0.612^∗∗∗^	-0.018	-0.146	0.085	-0.036	-0.051	

^∗^Y: consumer choice in favor of food products. ^∗∗^Correlation is significant at the level of 0.05 (bilateral). ^∗∗∗^Correlation is significant at the level of 0.01 (bilateral).

## Data Availability

Data available are on request. The data used to support the findings of this study are available from the corresponding author upon request (5792687@mail.ru).
